# Effect of Inductive Coil Shape on Sensing Performance of Linear Displacement Sensor Using Thin Inductive Coil and Pattern Guide

**DOI:** 10.3390/s111110522

**Published:** 2011-11-03

**Authors:** Norhisam Misron, Loo Qian Ying, Raja Nor Firdaus, Norrimah Abdullah, Nashiren Farzilah Mailah, Hiroyuki Wakiwaka

**Affiliations:** 1 Electrical and Electronic Engineering Department, Faculty of Engineering, Universiti Putra Malaysia, 43400 Serdang, Malaysia; E-Mails: looqian@gmail.com (L.Q.Y.); kashfi_@hotmail.com (R.N.F.); nashiren@eng.upm.edu.my (N.F.M.); 2 Malaysian France Institute, Universiti Kuala Lumpur, Section 14, Jln. Teras Jernang, 43650 Bandar Baru Bangi, Malaysia; E-Mail: norrimah@mfi.unikl.edu.my; 3 Faculty of Engineering, Shinshu University, 4-17-1 Wakasato, Nagano 380-8553, Japan; E-Mail: wakiwak@shinshu-u.ac.jp

**Keywords:** linear displacement sensor, inductive coil, pattern guide, meander shape, triangular type meander shape, square shape, circle shape, inductance, sensitivity, linearity

## Abstract

This paper discusses the effect of inductive coil shape on the sensing performance of a linear displacement sensor. The linear displacement sensor consists of a thin type inductive coil with a thin pattern guide, thus being suitable for tiny space applications. The position can be detected by measuring the inductance of the inductive coil. At each position due to the change in inductive coil area facing the pattern guide the value of inductance is different. Therefore, the objective of this research is to study various inductive coil pattern shapes and to propose the pattern that can achieve good sensing performance. Various shapes of meander, triangular type meander, square and circle shape with different turn number of inductive coils are examined in this study. The inductance is measured with the sensor sensitivity and linearity as a performance evaluation parameter of the sensor. In conclusion, each inductive coil shape has its own advantages and disadvantages. For instance, the circle shape inductive coil produces high sensitivity with a low linearity response. Meanwhile, the square shape inductive coil has a medium sensitivity with higher linearity.

## 1. Introduction

In modern industrial production processes the actual displacement of fast moving objects often needs to be detected and is ideally done without the use of any mechanical contact [1]. A variety of suitable sensors are available that can provide an output signal (voltage or current) proportional to the displacement of target and sensor. Magnetic sensor and optical sensor are the most commonly used sensors in industrial applications. In some robust working environments, the thin displacement sensor type is required due to the limited space in the system and harsh environment. For this type of application, the magnetic base detection sensor is suitable since it has no contact between the sensor head and sensor guide. For example, Ong *et al.* have studied in details the resonance frequency which can be applied for various kinds of detection using magnetic based sensors [2–4]. Meanwhile, optical sensors are not suitable since they are highly sensitive to the working environment, even though it provides good accuracy. There are many types of magnetic based displacement sensor that are being marketed and are researched recently. Some researchers are based on the capacitive concept for linear displacement sensor [5–7], and some of them use the concept of magnetostrictive linear position sensor [8,9]. Magnetostrictive delay line (MDL) technique and the eddy currents induced on a soft magnetic material also are introduced in [10,11].

In the magnetic based displacement sensor area, many researchers use planar versions with meander coils to detect planar displacements. The capacitive planar displacement sensor is designed and fabricated for measurement for a small displacement with very high accuracy. This sensor is a kind of linear encoder with an array of microelectrodes made by micromachining processes. The two patterned electrodes on the sensor substrates are assembled facing each other after being coated with a thin dielectric film. The inductive planar sensor is developed using printed circuit board (PCB) technology due to the low cost and detect even small displacements of less than 0.5 mm. Similarly, planar sensor structures are realized in either thick or thin film PCB technology [12–14]. In a planar displacement inductive sensor, the sensor is composed of two sensor elements. The first meander coil sensor element detects the vertical displacement while the second meander coil element detects the horizontal displacement. Combining the information from these two sensor elements, it is possible to determine displacement in a plane [15]. However, since the detection of the displacement depends on both meander coil elements the overall detection is limited to small displacements and is not suitable for applications that requires large displacements, especially of the single-axis motion sensory type.

A similar research regarding linear displacement sensor using a meander coil and pattern guide using an inductive concept is introduced in [16–19]. This kind of linear displacement sensor is almost identical to the planar type displacement sensor. However, only a single meander coil element is used whereas a solid structure named pattern guide is used as the second sensor element. In [16], the mathematical equation of the sensor output voltage is derived using a magnetic coupling method. In addition, the effect of input frequency on the output voltage is analyzed and compared with the measurement data.

In this paper a thin type inductive coil with a thin pattern guide is used as a linear displacement sensor. The position of the linear displacement sensor can be detected by measuring the coil inductance of the inductive coil. This linear displacement sensor exhibits superior advantages compared to other magnetic based sensor types rather than the optical based sensor type. Without a mechanical contact, it is good for usage with a longer lifetime and higher reliability. It has a simple structure due to its compact size and smaller thickness. These features allow the sensor to be embedded into the systems such as inside a linear motor for displacement applications. However, the sensitivity and linearity of this type of sensor can be further enhanced to achieve good sensory performance. One method of improvement is on the study of various inductive coil shapes and to propose a sensor structure that exhibits good accuracy that consequently reduces the signal processing time.

This paper presents the effect of inductive coil shape on the sensing performance of the linear displacement sensor. In this research, inductive coils with different coil turn numbers and various shapes such as meander shape, rectangular type meander shape, square shape and circle shape were fabricated and tested for performance evaluation. The paper proposes the possible pattern of inductive coil shape that be implemented in the linear displacement sensor using a meander coil and pattern guide.

## 2. Structure and Basic Principle of Linear Displacement Sensor

**Figure 1.**
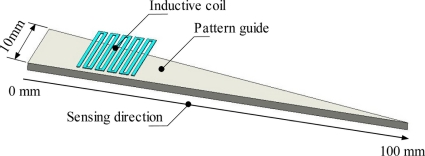
Structure of thin type of linear displacement sensor.

Figure 1 shows the structure of a thin type linear displacement sensor. The sensor consists of a thin type inductive coil with a thin pattern guide. The inductive coil is made from printed circuit boards with a very tiny (35 μm) copper layer. The printing circuit board was supplied by Instagraphic Products Ltd. The copper layer is then shaped with various inductive coil structures by using the same etching process of printed electronic circuit board making. The meander shape of inductive coil is represented in Figure 1, but in practical applications any shape can be used as long as the inductance value of inductive coil change depends on the positioning of the pattern guide. The pattern guide shape is a triangular structure pointing in the sensing direction. This pattern guide is made of ferromagnetic material so that a large significant difference on the inductance value occurred when the displacement is varied. Such ferromagnetic material is soft iron (SS400) with fine thickness up to 1 mm. With a thin inductive coil structure and a simpler pattern guide structure it is suitable for tiny space application such as a linear motor displacement sensor.

For typical industrial applications, the sensor must meet pre-set requirements in terms of reliability, ruggedness, measuring range, supply voltage range, output signal and EMC requirements. Optical sensors are one of the choices that are widely used in the modern industry because of their high accuracy. However in optical sensors, apart from being not suitable for operation in harsh environments, the accuracy decreases for any persistent obstacle in the detecting object. This major drawback is addressed with the proposed type of inductive coil based linear displacement sensor as it is highly insensitive to environmental influences such as oil, dirt and water.

**Figure 2.**
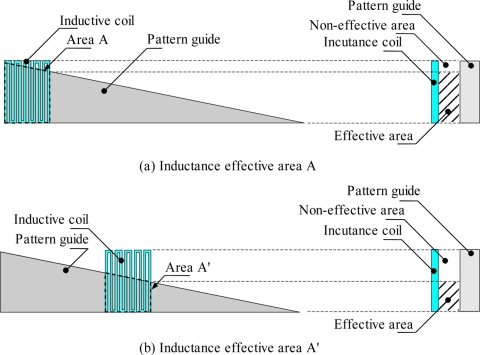
Comparison of inductance effective area at difference position of inductive coil.

This linear displacement sensor can detect the position based on the inductance value of the inductive coil in each position. At each position this value is different depending on the inductive coil area that faces the pattern guide effective area, as shown in Figure 2. The inductive effective area decreases as the inductive coil moves to the right side. It can be seen in Figure 2(a) that the effective area of the inductive coil is bigger compared to the position presented in Figure 2(b). The inductance value of an inductive coil is given in Equation (1), where the inductance value depends on the effective area *A* as discussed in [16]:

(1) $$L=\mu_{r}\mu_{0}nl_{c}A=\mu nl_{c}A$$

where *u_r_* is relative permeability of iron, *μ_0_* is relative permeability of air (4π × 10^−7^), *n* is number of turns of the coil, *l*_c_ is length of the coil and *A* is the effective cross sectional area of the coil. In the explanation of Figure 2, the meander type inductance coil type is used, but the inductance coil can be of any shape. Different inductive coil shapes give different sensing performance sensitivity and linearity characteristics. The study of the effect of shape on the sensing performance is the core objective of this paper:

The system controller of this displacement sensor is simple and is used to measure the inductance value of inductive coil and translate it to the displacement. As the controller continuously injects a high frequency sinusoidal voltage into the inductive coil, the current and the phase difference between voltage and current is measured to derive the inductance value at each position. Therefore, the frequency value and injected voltage are also important parameters for the sensor system.

## 3. Measurement and Evaluation of Various Shape of Inductive Coil

**Figure 3.**
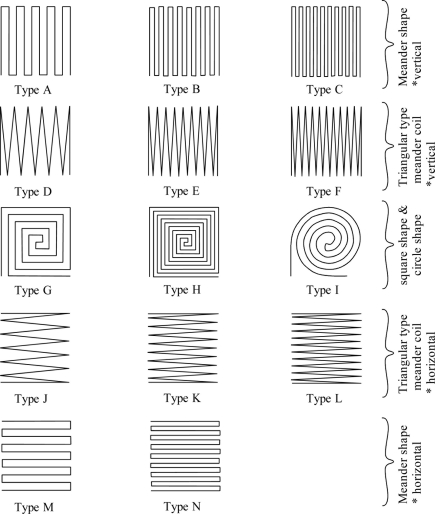
Various inductive coil shapes.

Figure 3 shows the various inductive coil shapes used in this study. Each type of inductive coil has the same external 10 mm × 10 mm area with 0.3 mm of coil width. The spaces between coils are distributed evenly and the distance is determined by coil turn numbers as long it is inside the fixed external area.

A, B, C, M and N are of the meander shape inductive type with both vertical and horizontal position settings with different coil turn numbers. Type A, B and C are positioned in a vertical position and Type M and N are positioned along a horizontal axis. Type A and M have 10 coil turns, Type B and N have 16 coils and Type C has 20 coils. D, E, F, J, K and L are of meander inductive triangular coil type. Type D, E and F are set vertically and Type J, K and L are set in the horizontal position. Type D and J have 12 coils, Type E and K have 18 coils and Type F and L has 22 coils. Type G and H inductive coils are of square shape with 5 and 9 coils, respectively. Type I is circle shape inductive coil with 5 coils.

**Figure 4.**
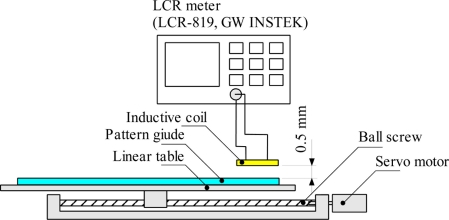
Measurement method of inductance at each position.

The inductance measurement at each position is obtained with the test bench set-up as shown in Figure 4. A servo motor with encoder connected to the ball screw of linear table is used to set the accurate position. The pattern guide is located at the top of linear table so that the position of the pattern guide can be controlled by the servo motor. In this experimental test bench setup the moving part is the pattern guide however in actual applications the inductive coil is the moving part. The inductive coil is held and fixed by jig stand on the top of pattern guide. The gap between the inductive coil and the pattern guide is 0.5 mm and it is set with a shim plate of 0.5 mm thickness.

The inductance values of the inductive coil measured for every 10.0 mm increment of pattern guide are measured for both forward and reverse direction using a LCR meter (LCR-819, GW INSTEK) where the frequency and the input voltage can be adjusted. The pattern guide moves up to the maximum displacement of 100 mm. Sensitivity and linearity of the sensor are used to evaluate the performance of inductive coils. Sensitivity is the ratio of a small change in electrical signal to a small change in physical signal and linearity is the maximum deviation of the sensor output from a Best Fit Straight Line (BFSL). A high linearity of the sensor performance reduces the signal processing time of a sensor system. The other sensor evaluation performance parameter such as noise, resolution, repeatability, hysteresis, accuracy, and dynamic range are discussed and examined as the sensor is connected to the complete system.

## 4. Inductance Characteristic Analysis of Various Shape of Inductive Coil

### 4.1. Effect of Operation Frequency

**Figure 5.**
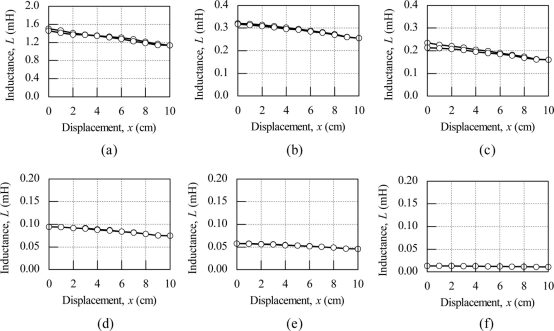
Effect of frequency on inductance characteristic for meander shape inductive coil (*V*_in_ = 1 V) **(a)** *f* = 5 kHz; **(b)** *f* = 10 kHz; **(c)** *f* = 15 kHz; **(d)** *f* = 20 kHz; **(e)** *f* = 25 kHz; **(f)** *f* = 50 kHz.

Figure 5 shows the inductance characteristics as the operational frequency varies from 5 kHz to 50 kHz for the meander shape inductive coil. The inductance value is inconsistent for less than 1 kHz and hence not examined. Since the same inductive coil is used for different operational frequencies, the only effect is on the sensor sensitivity is used as the same performance value of linearity is exhibited.

For the measurement of operation frequencies of 5 kHz and above, the inductance value decreases as the inductive coil position moves from 0 cm to 10 cm for all operational frequency ranges. The inductance values at the starting position of 0 cm decreases when the frequency is high. A big drop of 1.3 mH in the inductance value at the starting position can be seen between the operating frequencies of 5 kHz and 10 kHz. This big inductance value drop is because of the iron losses in the pattern guide due to the eddy current effect. For the other operational frequency ranges, the inductance value drop at the starting position is reasonably small.

The sensor sensitivity varies inversely with respect to the operation frequency. The sensor sensitivity for operation frequencies of 5 kHz and 10 kHz are 36 μH/cm and 6.5 μH/cm respectively. The sensor sensitivity for 5 kHz is 5.5 times higher than at the operational frequency 10 kHz. For operation frequencies above 10 kHz, the sensor sensitivity is below 5 μH/cm, which is very small.

### 4.2. Effect of Input Voltage

**Figure 6.**
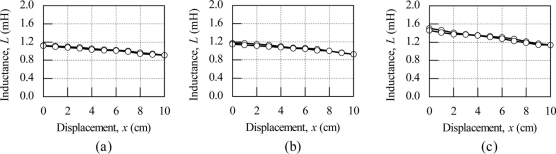
Effect of input voltage to the inductance characteristic for the meander shape inductive coil (*f* = 5 kHz) **(a)** *V*_in_ = 0.2 V **(b)** *V*_in_ =0.5 V **(c)** *V*_in_ = 1.0 V.

Figure 6 shows the effect of input voltage to the inductance characteristics of the meander shape inductive coil at an operational frequency of 5 kHz. The evaluation for the effect of input voltage also uses the same consideration as that of evaluation of operational frequency. The input voltage is varied for values of 0.2 V, 0.5 V and 1.0 V. For the input voltages of 0.2 V and 0.5 V the inductance value shows almost the same characteristics and sensitivity is better when the input voltage is 1.0 V. The sensitivity for 1.0 V is 37 μH/cm, which is 54% higher than the sensitivity for 0.5 V input voltage. Based on the above considerations on the effect of operation frequency and input voltage 5 kHz at 1.0 V is used to examine the effect of the inductive coil shape on the performance of the linear displacement sensor.

### 4.3. Effect of Inductive Coil Shape

**Figure 7.**
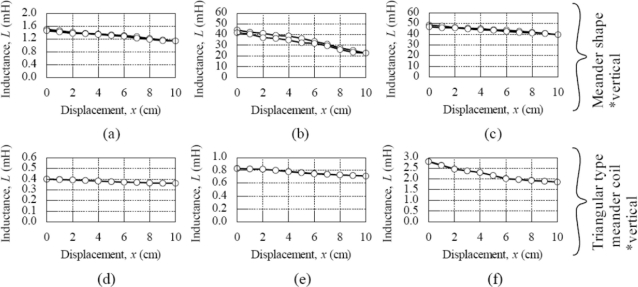

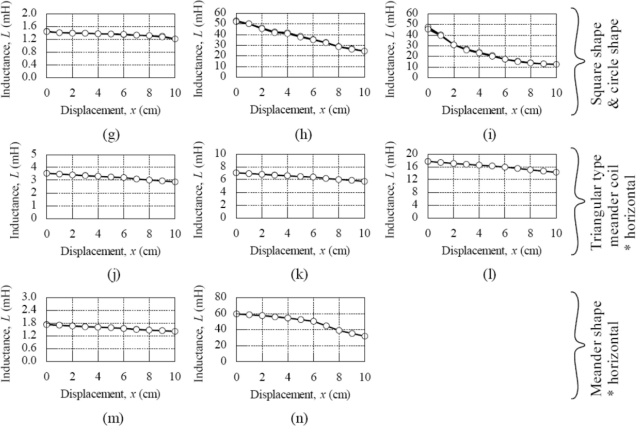
Inductance characteristic for various shapes of inductive coil (*f* = 5 kHz, *V*_in_ = 1 V) **(a)** Type A; **(b)** Type B; **(c)** Type C; **(d)** Type D; **(e)** Type E; **(f)** Type F; **(g)** Type G; **(h)** Type H; **(i)** Type I; **(j)** Type J; **(k)** Type K; **(l)** Type L; **(m)** Type M; **(n)** Type N.

**Figure 8.**
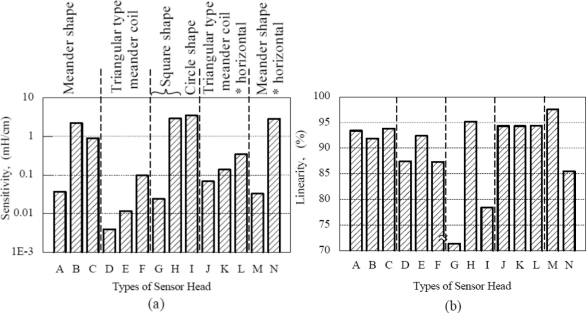
The sensitivity and linearity of various shapes of inductive coil **(a)** Various shape inductive coil sensitivity; **(b)** Various shape inductive coil linearity.

The characteristics of each type of inductive coil shape are discussed and presented in Figures 7 and 8. Figure 7 shows the inductance characteristic for various shapes of inductive coil and Figure 8 shows the sensitivity and linearity of various shapes of inductive coil. Type A, B and C have the same inductive coil shape but with different coil turn numbers. The average inductance value for Type A, B and C are 1.3 mH, 30 mH and 45 mH, respectively. The increase average inductance value is due to the larger coil turn numbers in Type B and C. The sensor sensitivity for Type B is 2.15 mH/cm, which is bigger compared to Type A and C, even though Type C has a high average inductance value. The sensor linearity for this type inductive coil shape is more than 90% for Type A, B and C, whereas the Type C shows a better sensor linearity value of 94%.

In the triangular type meander coils the average inductance value for Type D, E and F are 0.37 mH, 0.75 mH and 2.5 mH, respectively. The average inductance is smaller compared to the meander shape presented before, but it shows the same characteristic with meander shape, where the average inductance value is increasing due to the increase of coil numbers, but the increase of average inductance value is smaller compared to the meander shape. The sensor sensitivity for this type of inductive coils is smaller, with a value of less than 0.1 mH/cm. The linearity for Type D and F is about 87% and for Type E the linearity is 92%.

The square shape type G and H coils show a big difference in average inductance value, sensitivity and linearity with the same shape. The average inductance value for Type G is 1.3 mH and 38 mH for Type H. The sensitivity for type H is 2.8 mH/cm with 95%linearity when compared to Type G with sensitivity 0.24 mH/cm and 71% linearity. This big difference in average inductance value is due to the difference on coil turn numbers with Type G having 5 turns and Type H having 9 turns. It can be inferred that the turn number has a big effect on sensor performance for square shape inductive coils. Meanwhile, Type I has the highest sensor sensitivity of 3.4 mH/cm compared to other types of inductive coil used for this study, but the linearity for this shape is about 78%, which is less than the square shape inductive coils.

In order to study the effect of horizontal and vertical position, the comparison of triangular type meander shape coils of Type D, E, F and Type J, K, L is performed. The sensor sensitivity and linearity show some improvement in Type J, K and L. For example, Type L has 0.3 mH/cm sensitivity with 94% linearity, but Type F only has 0.1 mH/cm sensitivity and 87% linearity. This sensitivity improvement is still low compared to other inductive coil shapes. The comparison has also been made for meander shape inductive coil Type A, B and Type M, N. The comparisons on sensitivity for both types show similar results. The highest linearity of 97% is exhibited by Type M.

From the above discussion, the inductive coil with square shape gives a very good sensor performance effect. The sensitivity for this shape is 2.8 mH/cm with the linearity reaching up to 95%. The circle coil shape produces the highest sensitivity of 3.4 mH/cm, with the linearity being less than 80%. The triangular type meander coil shape has low sensitivity of less than 0.1 mH/cm, although the coil numbers are increased. The sensor performance for the meander coil shape can be improved by increasing the coil numbers, however large coil numbers can reduce the sensor sensitivity.

Sensor setting position does not have a high impact on the sensor performance whereas the sensor sensitivity for the triangular type meander shape coil showed some improvement, but still small compared to the square and circle shaped inductive coils. The meander shape also does not show any significant improvement in linearity even though the sensitivity exhibits almost the same result.

## 5. Conclusions

In this paper, the basic principle and the structure of the linear displacement sensor using a thin inductive coil and pattern guide is discussed. The effect of several inductive coil shapes such as the meander shape, rectangular type meander shape, square shape and circle shape are examined in this research study. All these various inductive coil shapes and coil turn numbers are fabricated and tested for evaluation of sensitivity and linearity performance. The inductive coil with square shape gives very good sensor performance effects, with sensitivity of 2.8 mH/cm and linearity reaching up to 95%. The circle coil shape produces the highest sensitivity in this study, with 3.4 mH/cm sensitivity, but the linearity is less than 80%. Even with the increase of coil numbers the triangular type meander coil shape has low sensitivity of less than 0.1 mH/cm. For the meander coil shape, the sensor performance can be improved by increasing the coil numbers, but at the same time we have to consider that large coil numbers can reduce the sensor sensitivity. It is inferred that the circle shape inductive coil can produce high sensitivity but with lower linearity values. Meanwhile, square shape inductive coils exhibit medium sensitivity with higher linearity characteristics. Therefore, a suitable inductive coil pattern needs to be appropriately selected based on the required application. The calculation of inductance would be presented in future work.
